# Alterations in the inflammatory cytokines and brain-derived neurotrophic factor contribute to depression-like phenotype after spared nerve injury: improvement by ketamine

**DOI:** 10.1038/s41598-017-03590-3

**Published:** 2017-06-09

**Authors:** Ze-Min Xie, Xing-Ming Wang, Ning Xu, Jing Wang, Wei Pan, Xiao-Hui Tang, Zhi-Qiang Zhou, Kenji Hashimoto, Jian-Jun Yang

**Affiliations:** 1 Department of Anesthesiology, Zhongda Hospital, Medical School, Southeast University, Nanjing, China; 2 Jiangsu Province Key Laboratory of Anesthesiology & Jiangsu Province Laboratory of Anesthetic and Analgesia Application Technology, Xuzhou Medicine University, Xuzhou, China; 3 Department of Anesthesiology, Jinling Hospital, School of Medicine, Nanjing University, Nanjing, China; 4 Division of Clinical Neuroscience, Chiba University Center for Forensic Mental Health, Chiba, Japan

## Abstract

Although pain is frequently accompanied with depression, little is known about the risk factors contributing to individual differences to the comorbidity of pain and depression. In this study, we examined whether cytokines and brain-derived neurotrophic factor (BDNF) might contribute to the individual differences in the development of neuropathic pain-induced depression. Rats were randomly subjected to spared nerved ligation (SNI) or sham surgery. The SNI rats were divided into two groups by the data from depression-related behavioral tests. Rats with depression-like phenotype displayed higher levels of pro-inflammatory cytokines (e.g., interleukin (IL)-1β, IL-6) as well as imbalance of pro/anti-inflammatory cytokines compared with rats without depression-like phenotype and sham-operated rats. Levels of BDNF in the prefrontal cortex of rats with depression-like phenotype were lower than those of rats without depression-like phenotype and sham-operated rats. A single dose of ketamine ameliorated depression-like behaviors in the rats with depression-like phenotype. Interestingly, higher serum levels of IL-1β and IL-6 in the rat with depression-like phenotype were normalized after a single dose of ketamine. These findings suggest that alterations in the inflammatory cytokines and BDNF might contribute to neuropathic pain-induced depression, and that serum cytokines may be predictable biomarkers for ketamine’s antidepressant actions.

## Introduction

Depression and pain are frequently comorbid in clinics. Epidemiological studies show that the prevalence of pain in depressed patients is up to 50%^1, 2^. Meanwhile, pain is a risk factor for depression, and the prevalence of painful patients with depression is around 30%^3, 4^. These epidemiological studies suggest that individual differences exist in the development of the comorbid pain and depression^2, 5–8^. Depression and pain share biological pathways, which have implications for the treatment of both concurrently. However, the precise mechanisms underlying the comorbidity of pain and depression are unknown. In addition, the possible predisposing factors for individual differences in this comorbidity are still poorly understood.

Neuro-immune system plays a critical role in the development of neuropathic pain^9, 10^ and depression^11–13^. Higher levels of pro-inflammatory cytokines (e.g., tumor necrosis factor (TNF)-α, interleukin (IL)-6 and IL-1β) in the central nervous system (CNS) contribute to the pathophysiology of neuropathic pain and depression^14–16^. In contrast, anti-inflammatory cytokines (e.g., IL-4 and IL-10) can influence nociceptive and depressive behaviors as a failure to counterbalance the over-expressed pro-inflammatory cytokines^17, 18^. Furthermore, imbalance of pro/anti-inflammatory cytokines is also observed in depressed patients^19^ and painful neuropathy patients^20^ who can be improved after antidepressant and analgesic treatment^21, 22^. Recent studies show that activated inflammatory response serves as a key mechanism for the comorbidity of pain and depression^23–26^. Furthermore, researchers shed more light on the mechanisms of individual differences in the pathogenesis of depression^27^. It is reported that vulnerability to depression might be closely related to exaggerated inflammatory response^28, 29^. Besides, the ratio of IL-6 to IL-10, which is the balance between proinflammatory (IL-6) and anti-inflammatory (IL-10) cytokines, is elevated in the individuals vulnerable to depression^30^. Therefore, the neuro-immune system may play a key role in vulnerability or resilience to inflammation (or stress). However, it is currently unknown how inflammation plays a role in the individual differences in the comorbidity of neuropathic pain and depression.

Brain-derived neurotrophic factor (BDNF) plays a key role in the pathogenesis of depression^31^, which is down-regulated in the prefrontal cortex (PFC) two weeks after neuropathic pain^32^. Alterations in the BDNF expression are implicated in the pathogenesis of depression and antidepressant mechanisms^33, 34^. We reported down-regulation of hippocampal BDNF in a chronic unpredictable stress^35^, inflammation^36^ and social defeat stress models^37, 38^. Several lines of evidence have suggested that BDNF can regulate the resilience to stress-induced depression-like phentype^36, 39–41^. However, little is known about the effect of BDNF on individual emotional response to peripheral nerve injury.

Ketamine, the *N*-methyl-D-aspartate (NMDA) receptors antagonist, showed rapid onset and long-lasting antidepressant effects in patients with treatment-resistant major depressive disorder (MDD)^42, 43^. A single sub-anesthetic dose (0.5 mg/kg) of ketamine relieves depressive symptoms in two-thirds of the MDD patients, lasting for over a week^44, 45^. Furthermore, ketamine has been reported to relieve pain-induced depression, which is independent of its antinociceptive effect^46, 47^. Although stimulation of neurogenesis and neuroplasticity via modulating of glutamatergic signaling as well as the mammalian target of rapamycin is involved antidepressant effects of ketamine^48^, it seems that inhibition of inflammatory response may account for antidepressant effects of ketamine^49, 50^. Interestingly, we also reported that serum IL-6 is a predictive biomarker for ketamine’s antidepressant effect in patients with MDD^51, 52^. However, the roles of cytokines in ketamine’s antidepressant response have not been explored in rodent models of the comorbid neuropathic pain and depression.

The purpose of the present study was undertaken to examine whether cytokines and BDNF might contribute to the individual differences in rats after spared nerve injury (SNI). Furthermore, we examined whether ketamine could improve the comorbid neuropathic pain and depression after SNI.

## Results

### SNI induced long-lasting mechanical hyperalgesia and depression-like behaviors

**Figure 1:**
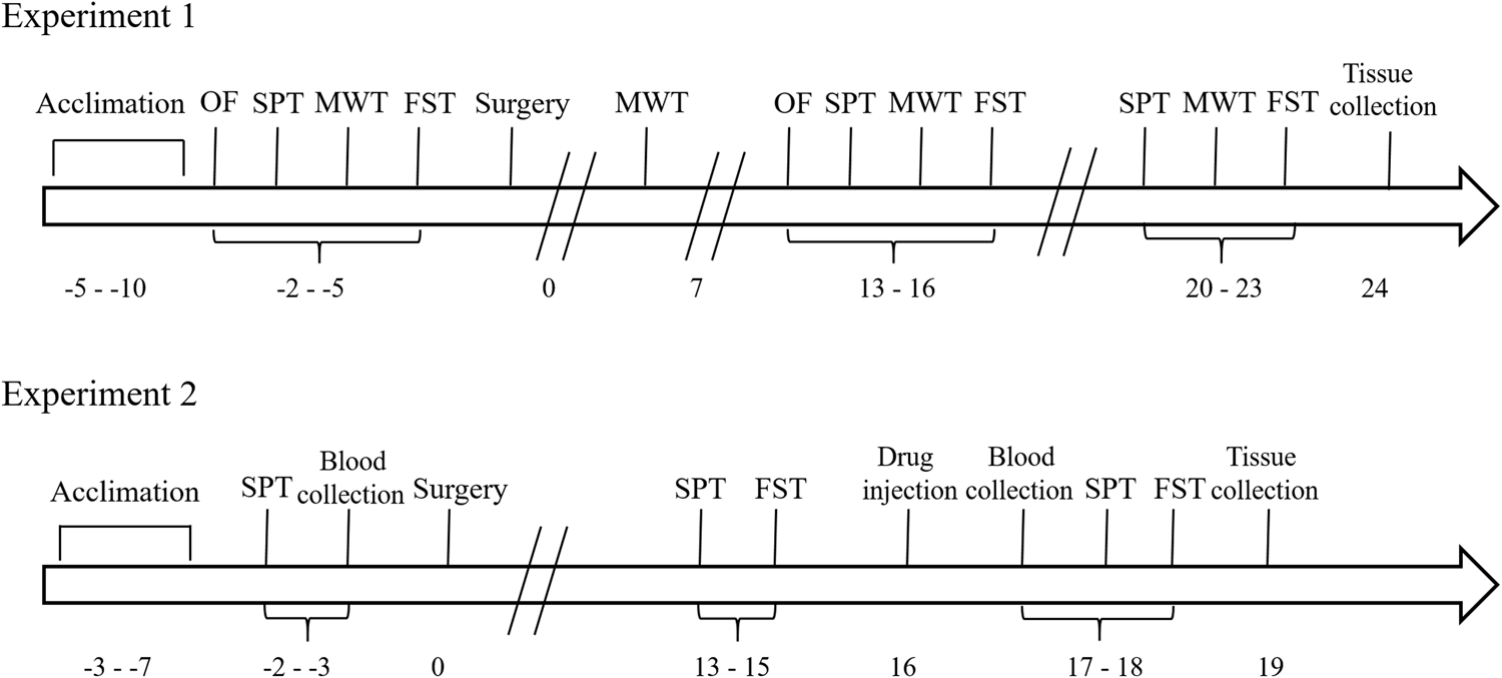
Schedule of behavioral tests and drug treatment.

In the experiment 1 (Fig. 1), SNI surgery induced mechanical hyperalgesia compared with sham surgery on days 7, 14 and 21 after surgery (*P* < 0.001) while the baseline mechanical threshold exhibited no difference between them (Fig. S1a). Furthermore, SNI rats displayed less weight gain compared with sham-operated rats on days 14 and 21 after surgery (*P* < 0.01; Fig. S1b). In the SPT, SNI rats showed decreased sucrose preference on days 14 (*P* < 0.01, *vs* baseline and sham-operated rats) and 21 (*P* < 0.05, *vs* baseline) after surgery (Fig. S1c), although total fluid intake was similar in the two groups (Fig. S1d). In the FST, the immobility time of SNI rats on days 14 and 21 after SNI surgery was higher than sham-operated rats (*P* < 0.05; Fig. S1e). Total distance traveled in the open field had no difference between sham-operated and SNI rats before and after surgery, which indicated that the locomotor ability was not affected by the surgery (Fig. S1f). These findings show that SNI produced mechanical hyperalgesia and depression-like behaviors in rats, consistent with previous report^46^.

### Classification of SNI rats with or without depression-like phenotype

**Figure 2:**
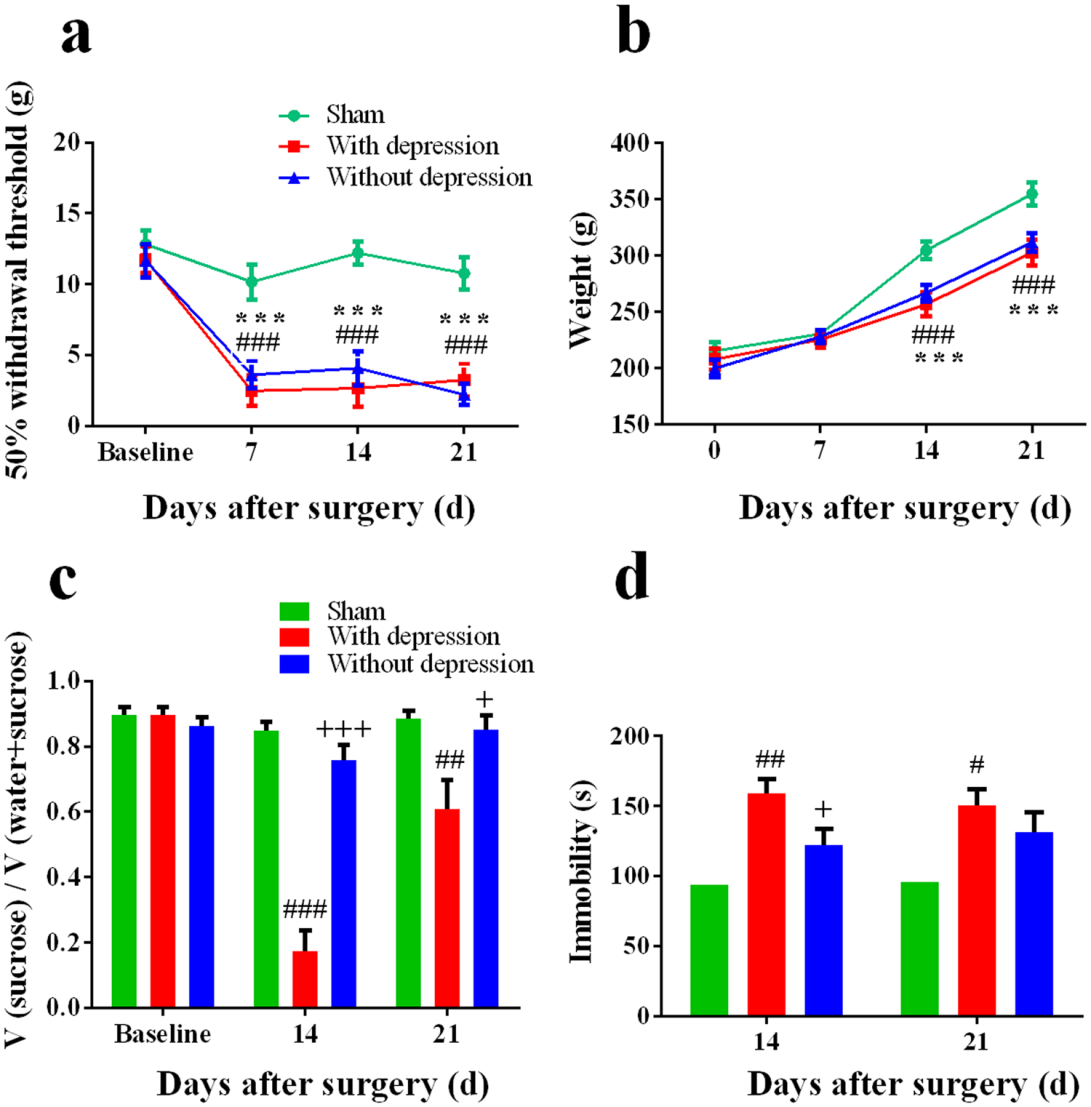
Behavior assessments of rats with or without depression-like phenotype. (**a**) In the MWT, both rats with or without depression-like phenotype showed mechanical hyperalgesia compared with sham group. There was no difference between two groups. (**b**) Both rats with or without depression-like phenotype showed less weight gain compared with sham group. There was no difference between two groups. (**c**) In the SPT, rats with depression-like phenotype displayed reduced sucrose preference compared with the sham-operated rats and rats without depression-like phenotype on days 14 and 21 after surgery. (**d**) In the FST, rats with depression-like phenotype (n = 13) displayed increased immobility time compared with sham-operated rats (n = 14) and rats without depression-like phenotype (n = 19). ^#^ *P* < 0.05, ^##^ *P* < 0.01 and ^###^ *P* < 0.001 for rats with depression-like phenotype vs sham group; *P < 0.05 and ***P < 0.001 for rats without depression-like phenotype vs sham group; ^+^ *P* < 0.05 and ^+++^ *P* < 0.001 for rats with depression-like phenotype vs rats without depression-like phenotype.

We applied hierarchical cluster analysis to classify the SNI rats into two clusters. In order to distinguish between the depression and not-depression phenotypes, the results in the SPT and FST were analyzed by two-way ANOVA. Then, 13 of 32 (40%) rats showing depression-like phenotypes were regarded as “rats with depression-like phenotype” (with depression), and the others were “rats without depression-like phenotype” (without depression) (Fig. S2). Both rats with or without depression-like phenotype showed mechanical hyperalgesia and decreased weight gain on days 14 and 21 after SNI surgery compared with sham-operated rats (*P* < 0.001), although there was no difference in mechanical thresholds or weight gain between two SNI groups (Fig. 2a,b). In the SPT, rats with depression-like phenotype displayed reduced sucrose preference compared with sham-operated rats and rats without depression-like phenotype on days 14 (*P* < 0.001) and 21 (*P* < 0.05) (Fig. 2c). In the FST, rats with depression-like phenotype had increased immobility time on day 14 (*P* < 0.05, *vs* sham-operated and rats without depression-like phenotype, respectively) or 21 (*P* < 0.05, *vs* sham-operated rats) after surgery (Fig. 2d).

### Different levels of cytokines and BDNF in the prefrontal cortex (PFC) in rats with or without depression-like phenotype

**Figure 3:**
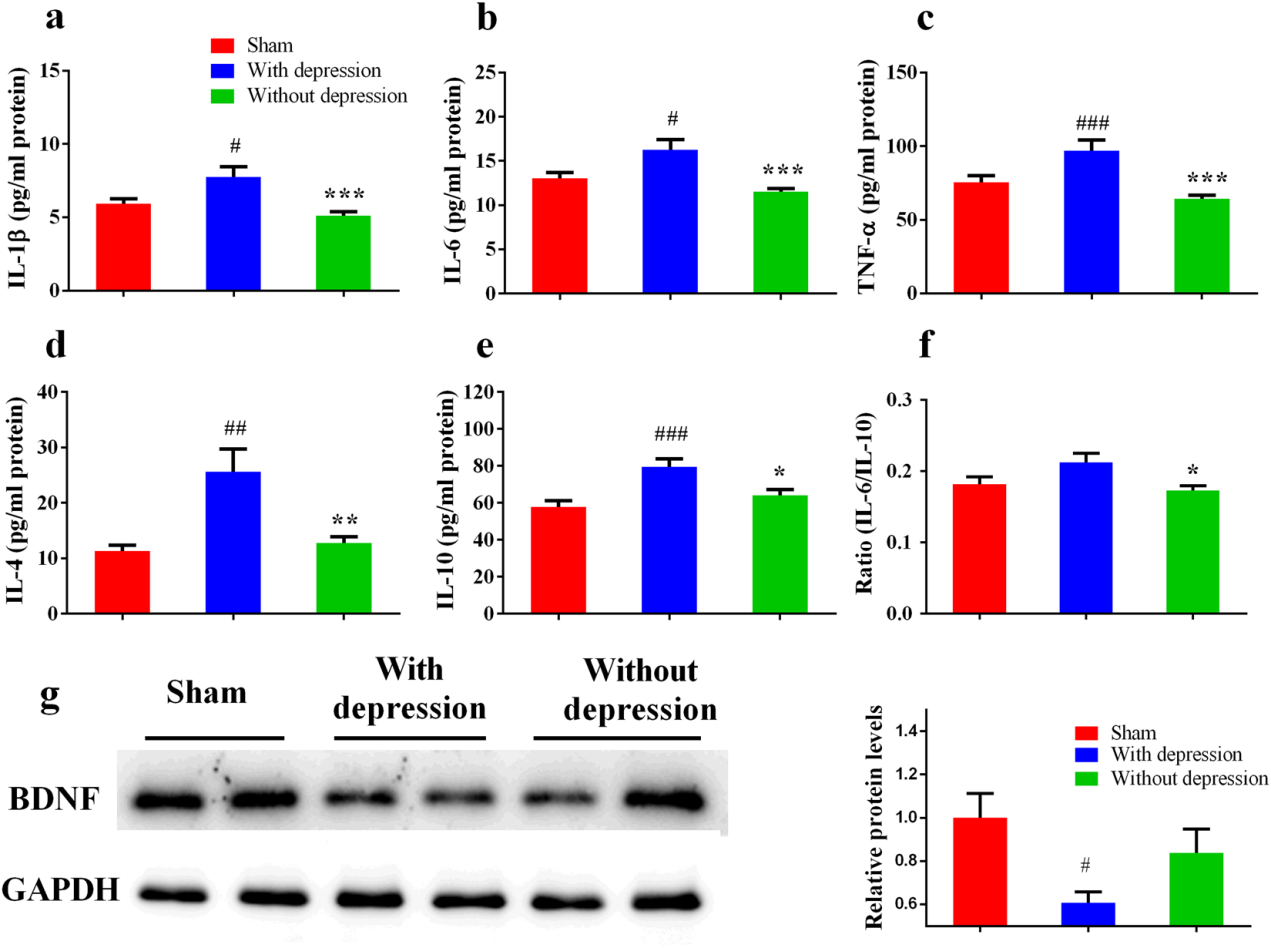
Expression of inflammatory cytokines in the PFC. (**a**) The levels of IL-1β in the PFC from rats with depression-like phenotype were higher than sham-operated rats and rats without depression-like phenotype. (**b**) The levels of IL-6 in the PFC from rats with depression-like phenotype were higher than sham-operated rats and rats without depression-like phenotype. (**c**) The levels of TNF-α in the PFC from rats with depression-like phenotype were higher than sham-operated rats and rats without depression-like phenotype. (**d**) The levels of IL-4 in the PFC from rats with depression-like phenotype were higher than sham-operated rats and rats without depression-like phenotype. (**e**) The levels of IL-10 in the PFC from rats with depression-like phenotype were higher than sham-operated rats and rats without depression-like phenotype. (**f**) There was no difference of the ratio of IL-6 to IL-10 between rats with depression-like phenotype and sham-operated rats. The ratio of IL-6 to IL-10 in the rats without depression-like phenotype was lower than rats with depression-like phenotype. (**g**) Western blot analysis of BDNF in the PFC after SNI surgery. ^#^ *P* < 0.05, ^##^ *P* < 0.01 and ^###^ *P* < 0.001 vs sham group; *P < 0.05, **P < 0.01 and ***P < 0.001 vs rats without depression-like phenotype. Each group, n = 6 or 7 for Elisa and n = 4 for western blot.

To evaluate the role of neuroinflammation in the individual differences of the comorbidity, we measured the levels of proinflammatory cytokines (IL-1β, IL-6 and TNF-α) and anti-inflammatory cytokines (IL-4, IL-10) in the prefrontal cortex (PFC) using ELISA. Rats with depression-like phenotype displayed increased levels of cytokines (IL-1β, IL-6, TNF-α, IL-4 and IL-10) in the PFC compared with sham-operated and rats without depression-like phenotype (*P* < 0.05) on day 21 after SNI surgery. In addition, the levels of these cytokines showed no difference between rats without depression-like phenotype and sham-operated rats (Fig. 3a–e).

To ascertain the balance between pro- and anti-inflammation, we assessed the ratio of IL-6 to IL-10 in the PFC. The ratio of rats with depression-like phenotype was higher than that of rats without depression-like phenotype (*P* < 0.05; Fig. 3f).

To determine the role BDNF in the individual differences of the comorbidity, we performed Western blot analysis of BDNF in the PFC. Levels of BDNF in the PFC of rats with depression-like phenotype were significantly lower than those of rats without depression-like phenotype and sham-operated rats (*P* < 0.05; Fig. 3g).

### Individual differences of ketamine’s antidepressant effect on day 14 after SNI surgery

**Figure 4:**
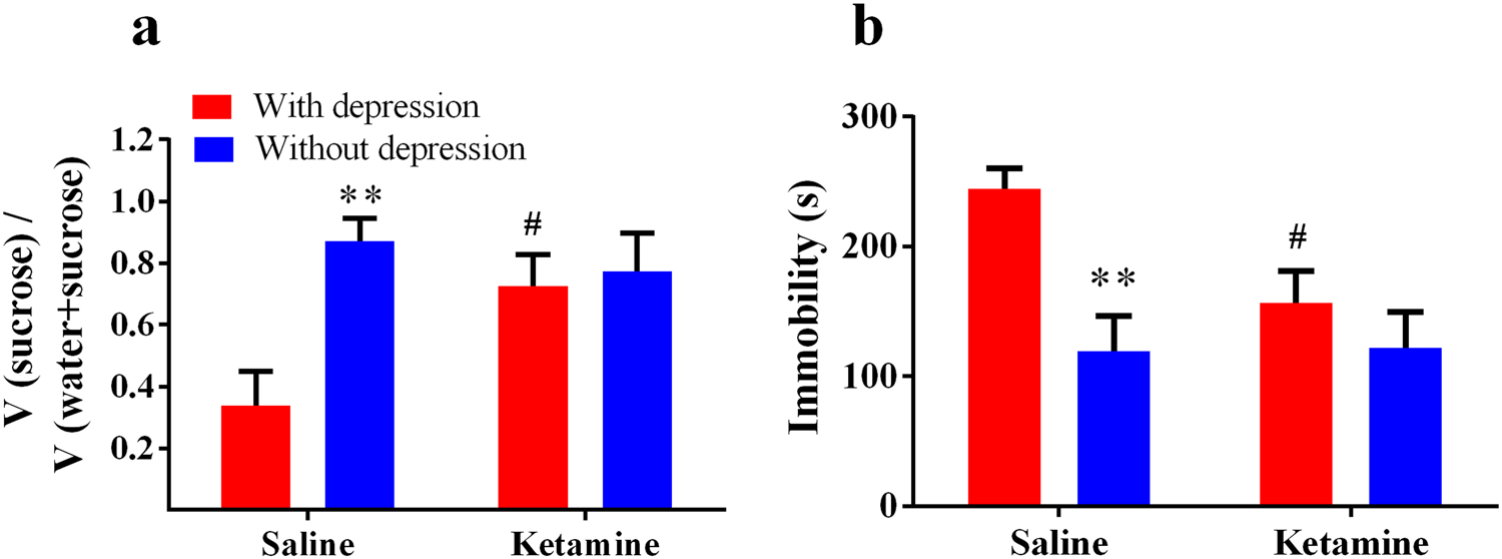
Ketamine’s antidepressant effects on day 14 after SNI surgery. (**a**) In the SPT, rats with depression-like phenotype displayed decreased sucrose preference than rats without depression-like phenotype rats after saline injection. A single injection of ketamine (20 mg/kg) attenuated decreased sucrose preference in the rats with depression-like phenotype. (**b**) In the FST, rats with depression-like phenotype displayed longer immobility time than rats without depression-like phenotype after saline injection. A single injection of ketamine (20 mg/kg) attenuated increased immobility time in the rats with depression-like phenotype. **P < 0.01 for rats without depression-like phenotype vs rats with depression-like phenotype after saline treatment. ^#^ *P* < 0.01 for ketamine treatment vs saline treatment in rats with depression-like phenotype.

In the experiment 2 (Fig. 1), we examined the effects of ketamine on the comorbidity after SNI surgery. Using the equation from discrimination analysis, 12 of 30 (40%) SNI rats were classified as “with depression-like phenotype”, and the others were regarded as “without depression-like phenotype”. Saline or ketamine (20 mg/kg) was injected into rats. In the SPT, rats with depression-like phenotype displayed less sucrose preference than rats without depression-like phenotype (*P* < 0.05, Fig. 4a). A single dose of ketamine (20 mg/kg) significantly increased decreased sucrose preference of rats with depression-like phenotype (*P* < 0.05, Fig. 4a). In contrast, ketamine displayed no difference in sucrose preference of rats without depression-like phenotype (Fig. 4a). In the FST, rats with depression-like phenotype displayed longer immobility time than rats without depression-like phenotype (*P* < 0.05, Fig. 4b). A single dose of ketamine (20 mg/kg) significantly decreased an increased immobility time of rats with depression-like phenotype (*P* < 0.05, Fig. 4b). In contrast, ketamine displayed no difference in immobility time of rats without depression-like phenotype (*P* < 0.05, Fig. 4b). These results show that ketamine can show a rapid antidepressant effect in rats with depression-like phenotype, but not rats without depression-like phenotype, on day14 after SNI surgery.

### Role of serum cytokines in ketamine’s antidepressant efficacy

**Figure 5:**
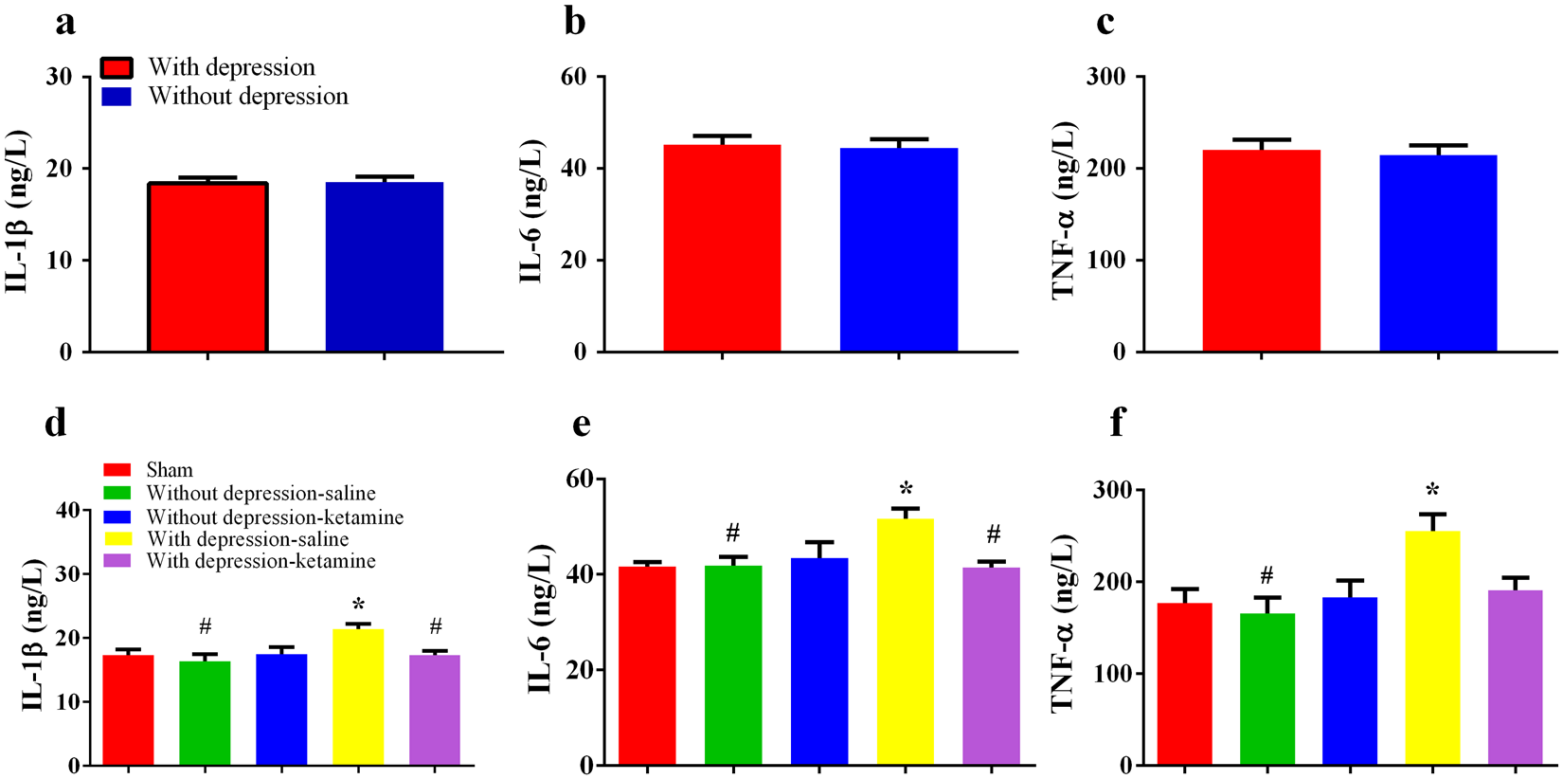
Serum levels of pro-inflammatory cytokines. Serum levels of (**a**) IL-1β, (**b**) IL-6 and (**c**) TNF-α at baseline were not different between rats with or without depression-like phenotype. Saline-treated rats with depression-like phenotype displayed higher serum levels of (**d**) IL-1β, (**e**) IL-6 and (**f**) TNF-α compared with sham-operated rats and rats without depression-like phenotype. A single dose of ketamine (20 mg/kg) reduced the serum levels of IL-1β and IL-6 in the rats with depression-like phenotype. *P < 0.05 vs with sham group, ^#^ *P* <0.05 vs depression-saline group.

There were no differences of serum baseline cytokines between rats with or without depression-like phenotype (Fig. 5a–c). To determine the role of serum cytokines in antidepressant effect of ketamine, we measured serum levels of cytokines IL-1β, IL-6 and TNF-α 3 days after a single dose of ketamine (20 mg/kg). Rats with depression-like phenotype showed higher serum levels of IL-1β, IL-6 and TNF-α compared with sham-operated group (*P* < 0.05, Fig. 5d–f). Furthermore, ketamine could decrease serum levels of IL-1β and IL-6 in rats with depression-like phenotype compared with saline treatment (*P* < 0.05, Fig. 5d,e). Rats with depression-like phenotype displayed higher serum levels of IL-1β, IL-6 and TNF-α than rats without depression-like phenotype (*P* < 0.05, Fig. 5d–f).

**Figure 6:**
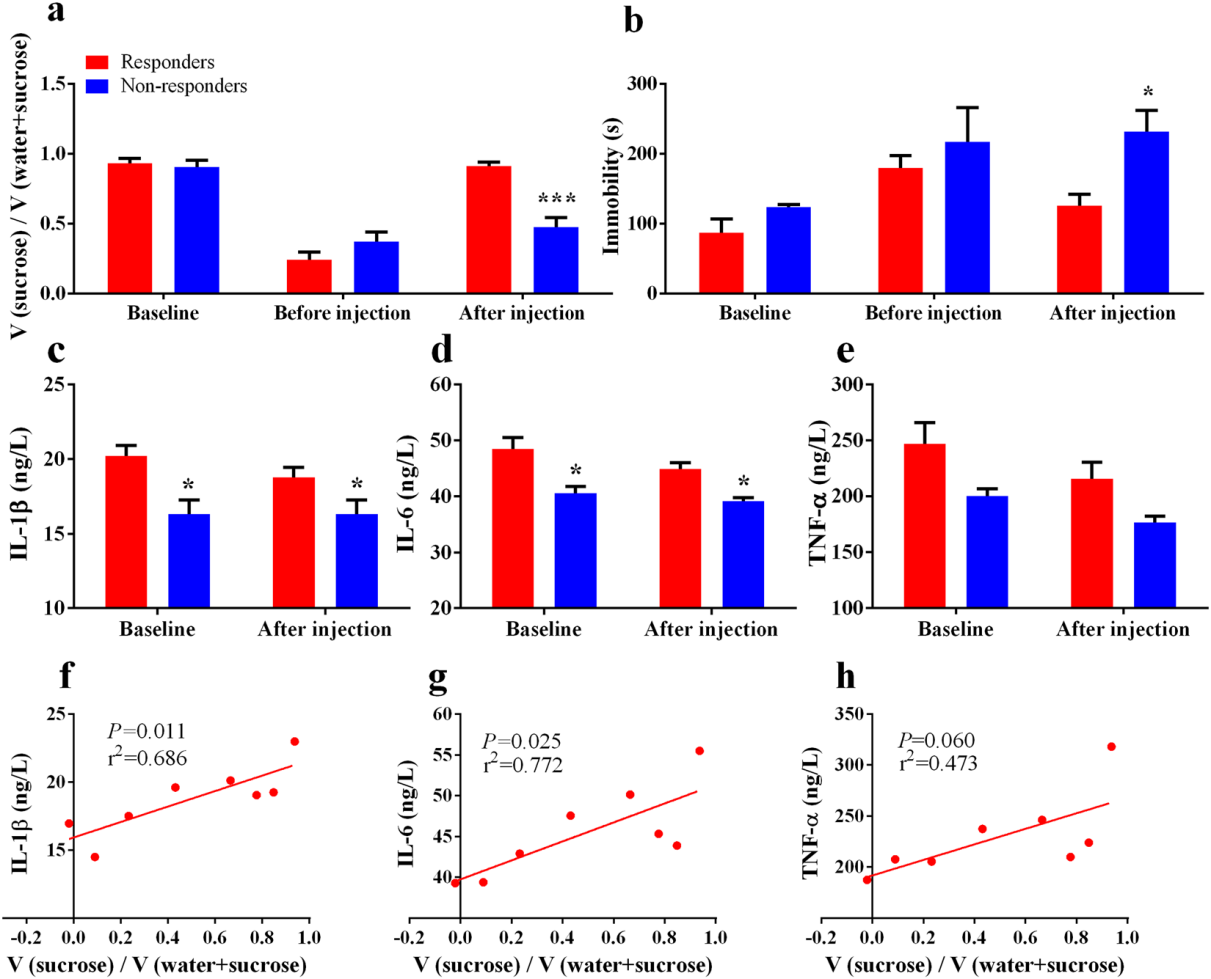
Serum pro-inflammatory cytokines for ketamine’s antidepressant actions. (**a**,**b**) Ketamine non-responder group showed decreased sucrose preference and increased immobility time after a single dose of ketamine (20 mg/kg). (**c–e**) Ketamine non-responder group showed decreased serum levels of IL-1β and IL-6 at baseline and after ketamine injection. There was no difference of serum levels of TNF-α between ketamine-responder group and non-responder group at baseline and after ketamine injection. (**f**,**g**) There were positive correlations between the changes of sucrose preference after ketamine injection and serum levels of IL-1β and IL-6 or TNF-α at baseline. (**h**) There was no correlation between the changes of sucrose preference after ketamine injection and serum levels of TNF-α at baseline. ^*^ *P* < 0.01 and ^***^ *P* < 0.001 vs ketamine-responder group.

To determine the ketamine’s response in rats with depression-like phenotype, discrimination analysis was applied to differentiate between responder group and non-responder group. Three of 8 rats with depression-like phenotype were classified as “non-responder group”, and the other 5 rats were regarded as “responder group”. Responder group displayed increased sucrose preference in the SPT (*P* < 0.001) and decreased immobility time (*P* < 0.05) in the FST after a single dose of ketamine compared with non-responder group. There was no difference between two groups at baseline and before ketamine injection (Fig. 6a,b).

Responder group showed higher serum levels of IL-1β and IL-6 than non-responder group at baseline and after ketamine injection (*P* < 0.05). Responder group had similar serum levels of IL-1β and IL-6 between baseline and after ketamine injection (Fig. 6c,d). No difference of TNF-α levels was observed at baseline and after ketamine injection between responders and non-responders (Fig. 6e).

To validate whether serum levels of cytokines at baseline can predict ketamine’s antidepressant action, we conducted a correlation analysis between the changes of sucrose preference after ketamine injection and serum levels of cytokines at baseline. Interestingly, there were positive correlations between the changes of sucrose preference and the baseline levels of serum IL-1β and IL-6 (Fig. 6f, r^2^ = 0.686, *P* = 0.011; Fig. 6g, r^2^ = 0.772, P = 0.025). However, there was no correlation between the changes of sucrose preference and serum levels of TNF-α (Fig. 6h, r^2^ = 0.473, P = 0.060).

## Discussion

The major findings of this study are as follows: Although SNI rats suffered near identical nociceptive damage, there were rats with or without depression-like phenotype. Rats with depression-like phenotype are susceptible, but rats without depression-like phenotype are resilience to SNI. Rats with depression-like phenotype had elevated neuroinflammtory response and imbalance of pro/anti-inflammatory compared with rats without depression-like phenotype and sham-operated rats. Tissue levels of BDNF in the PFC from rats with depression-like phenotype were lower than those of rats without depression-like phnotype and sham-operated rats. Furthermore, a subanethetic single dose of ketamine ameliorated depression-like behaviors in the rats with depression-like phenotype on day 14 after SNI surgery. Interestingly, higher serum levels of IL-1β and IL-6 may accout for ketamine’s antidepressant effect in the rat with depression-like phenotype after SNI. To our best knowledge, this is the first study showing the role of neuroinflammation and BDNF for individual differences of depression induced after neuropathic pain. In addition, this is also the first study showing the role of serum IL-1β and IL-6 in the individual differences of ketamine’s antidepressant response in the comorbidity of neuropahtic pain and depression in rodents.

In the preclinical studies, hyperalgesia rats were highly heterogeneous in depression-related behaviors. Keay and colleagues reported that chronic constriction injury (CCI) induces a subgroup (around 30%) of rats which were altered in dominant behavior^7^ and sleep-wake cycles^53^ using resident-intruder social interaction and sleep-wake analysis. In this study, the hierarchical cluster analysis divided SNI rats into two clusters: one group (40%, ‘depression-like phenotype’) with increased immobility time in the FST and reduced sucrose preference in the SPT, the other group (60%, ‘without depression-like phenotype’) with similar immobility time and sucrose preference compared with sham-operated rats. Previous clinical studies demonstrate that the incidence of comorbid chronic pain and depression is around 30% to 50%^1–4^, which is in line with the present study. In this study, we found that rats with or without depression-like phenotype showed similar mechanical withdrawal threshold, suggesting that the alterations in mood-related behaviors were independent of the degree of nociceptive damage, consistent with previous studies^7, 53–55^.

Elevated inflammatory response may account for the different alterations in mood-related behaviors induced by SNI. It is well recognized that neuroimmune alterations play vital roles in the course of pain and depression^9, 11, 55^. Nerve injury results in mood disturbance associated with up-regulation of IL-1β^24^, IL-6^26^ and TNF-α^56^ in the PFC, and inhibition of inflammation rescues mood disturbance. Inflammatory signaling is triggered after peripheral nerve injury, which projects to the several supraspinal regions (including the PFC) via neural, humoral or cellular pathways^57^. In this study, we observed increased levels of proinflammatory cytokines of IL-6, IL-1β, TNF-α as well as anti-inflammatory cytokines of IL-4 and IL-10 in the rats with depression-like phenotype, but not rats without depression-like phenotype. Wood *et al*.^30^ reported that exaggerated inflammatory response may elicit individual differences in risks for stress-induced depression. Pro-inflammatory (IL-1β) genetic variation and associated higher IL-1β expression increase risks for stress-induced depression^58^. Taken together, it is likely that elevated inflammatory response might contribute to vulnerability to depression-like phenotype induced by neuropathic pain.

The imbalance between pro- and anti-inflammatory cytokines may contribute to mood disorders^59, 60^. In this study, the ratio of IL-6 to IL-10 in the PFC of rats with depression-like phenotype was higher than rats without depression-like phenotype. A study using 66 patients demonstrated that depression group had higher ratio of IL-6 to IL-10 than control group^61^. Furthermore, it is also shown that increased ratio of pro- to anti-inflammatory is detected in animals with stress vulnerability^27, 30^. Additionally, the M1 and M2 polarization state of microglia, which is associated with increased pro- and anti-inflammatory cytokines, respectively, might be involved in the development of depression and neuropathic pain^26, 62^. Enhanced IL-6/IL-10 could be explained by more M1 than M2 phenotype of microglia, which would produce more pro-inflammatory cytokines and lead to a sick state. Therefore, it seems that the imbalance of pro/anti-inflammatory response might be an important factor for individual differences of the comorbidity.

We found the decreased levels of BDNF in the PFC on day 21 after SNI, consistent with the previous study^32^. Furthermore, we also found lower expression of BDNF in the rats with depression-like phenotype than rats without depression-like phenotype. Low levels of BDNF in the PFC are associated with the development of depression-like phenotype in rodents^36, 41, 63, 64^. BDNF plays a key role in promoting neurogenesis, resilience to damage, as well as neural plasticity^31, 65, 66^. In addition, pro-inflammatory cytokines may damage neuronal structure and function by suppression of BDNF^67, 68^. By contrast, anti-inflammatory cytokine IL-4 promotes glia to release BDNF^69^. Together, it is likely that reduced levels of BDNF in the PFC might be implicated in the depression vulnerability in the comorbid neuropathic pain and depression.

Ketamine is well known to improve both depression and neuropathic pain. Sub-anesthetic ketamine relives depression-like behaviors^35^ and the dose of ketamine above 25 mg/kg is necessary for analgesia^70^. A single dose (20 mg/kg) of ketamine ameliorated neuropathic pain-induced depression but had no analgesia effect^46^. In addition, ketamine (20 mg/kg) showed antidepressant effects in rat learned helplessness model of depression^37^. Although some studies have focused on ketamine’s antidepressant effects in the comorbidity of pain and depression^46, 47, 51^, there was no study showing individual differences of ketamine’s antidepressant effects in the comorbidity of pain and depression. Sub-anesthetic dose of ketamine increased social interaction of stress susceptible, but not stress resilient rats, after social defeat stress^71^, consistent with our study. In this study, we found that ketamine could attenuate decreased serum levels of IL-1β and IL-6, but not TNF-α level, in rats with depression-like phenotype. Moreover, we observed that 3 of 8 depression rats regarded as ketamine non-responder group displayed lower serum levels of IL-1β and IL-6. Interestingly, we reported that ketamine responders had higher levels of IL-1β and IL-6 at baseline compared with non-responders in the treatment-resistant patients with MDD^51^. Ketamine’s antidepressant effects may dependent on regulation of Th2-mediated humoral immunity. The imbalance between Th1 (e.g., TNF-α) and Th2 (e.g., IL-6) immunity may play vital role in the pathophysiology of MDD since the higher levels of IL-6 might predict ketamine’s antidepressant efficacy^52^. In this study, we found lower serum levels of IL-1β and IL-6 in non-responders at baseline, consistent with report using depressed patients^51^.

In this study, we found that SNI rats with depression-like phenotype showed higher inflammatory cytokines than SNI rats without depression-like phenotype. Previously, it was reported that peripheral IL-6 may contribute to resilience versus susceptible to inescapable stress^72^. Thus, it is likely that elevated inflammatory responses may play a role in the depression-like phenotype after SNI. However, the precise mechanisms underlying the relationship between elevated inflammatory responses and susceptibility to SNI are currently unknown. Interestingly, we reported that alterations in the composition of gut-microbiota may confer resilience to chronic social defeat stress^73^. Given the key role of gut-microbiota-brain axis in the psychiatric and neurological disorders^74, 75^, it is likely that alterations in the gut-microbiota may contribute to susceptibility to SNI surgery. Further detailed studies on the role of gut-microbiota in SNI model will be needed.

In the preliminary experiments, we performed the novelty-suppressed feeding (NSF) test to assess the depressive symptoms^76, 77^. However, we did not find any difference in the latency to feed after ketamine injection. Although the reasons underlying the negative effect of ketamine are unknown, the experimental protocols (e.g., different animal models, experimental apparatus, dose of ketamine, and observational protocol) may contribute to the negative result. In addition, we did not perform the OF test after ketamine injection in the experiment 2 because the total distance, indicating the motor function in the OF test, is not affected by a single sub-anesthetic dose of ketamine in our previous studies^35, 47, 64^ and other reports^46, 78^. Together, it seems that ketamine may not have beneficial effects on motor function deficits after SNI surgery. Nonetheless, further studies on the effects of ketamine in the SNI surgery model are needed.

In conclusion, the present study suggests that elevated inflammatory response, especially pro-inflammatory cytokines, and reduced levels of BDNF in the PFC play key roles in individual differences of comorbid neuropathic pain and depression. Furthermore, serum levels of IL-1β and IL-6 at baseline may predict ketamine’s antidepressant effects in pain-induced depression. Therefore, inflammatory cytokines may play a role in the depression vulnerability and ketamine’s antidepressant efficacy in the comorbid neuropathic pain and depression.

## Materials and Methods

### Animals

Male Sprague-Dawley rats (6–8 weeks, 150–180 g) were purchased from the Animal Center of Jingling Hospital, Nanjing, China. All procedures in this study were performed in accordance with the Guideline for the Care and Use of Laboratory Animals from the National Institutes of Health, USA. The protocol was approved by the Southeast University Institutional Animal Care and Use Committee. Rats were housed with water and food *ad libitum* under a 12-h light/dark cycle in a temperature-controlled room at 24 ± 1 °C.

### Experimental design

Experiment 1 (Fig. 1) Rats were acclimated to environment and sucrose intake for 5 days. We performed the OF, SPT and MWT from days 2 to 5 before surgery (baselines), the OF, SPT, MWT and FST from days 13 to 16 after surgery (test phase 1), and the SPT, MWT and FST from days 20 to 23 after surgery (test phase 2). Then the brain tissues were harvested and the PFC tissues were dissected. In addition, rats were weighted each week.

Experiment 2 (Fig. 1) rats were acclimated to environment and sucrose intake for 5 days. The SPT was performed and blood samples from orbital venous sinus were obtained on day 2 before surgery, and the SPT and FST were conducted from days 13 to 15 after surgery. A single dose of ketamine (20 mg/kg in 1 ml, Hengrui Pharmaceutical Company, Jiangsu, China) or the same volume of saline 1 ml was intraperitoneally injected on day 16 after surgery. Blood samples from orbital venous sinus were collected 24 h after intraperitoneal injection. The SPT and FST were performed to investigate the antidepressant effect of ketamine 48 h after intraperitoneal injection. Then, the PFC tissues were collected.

### Spared Nerve Injury (SNI)

The SNI surgery was performed as previously described^79^. With the anesthesia of intraperitoneal injection of 2% sodium pentobarbital (60 mg/kg, Sigma, St Louise, MO, USA), rats were incised from skin on the right thigh and bluntly dissected biceps femoris muscle. Sciatic nerve and its three terminal branches were exposed: the sural, common peroneal and tibial nerves. The common peroneal and tibial nerves were tight-ligated with 4–0 silk and sectioned the distal to the ligation. In the sham surgery, rats only received blunt dissection of the muscle and exposed nerve, but without ligation and cut of the nerve. Muscle and skin layers were then closed by 4–0 silk.

### Mechanical withdrawal test (MWT)

A traditional Dixon up-down method with von Frey filaments was used to measure mechanical allodynia^80, 81^. Before the test, rats were individually placed into transparent plexiglass chambers over a mesh table and habituated for about 20 min. Von Frey filaments started with 2.0 g were applied to the lateral 1/3 of right paws (in the distribution of the sural nerve) of rats. The maximum score was 15.10 g, and the minimum was 0.25 g. Paw withdrawals in response to the stimuli were considered positive, and no withdrawal was negative. Depending on the positive or negative result, descending or ascending intensity of subsequent filament was applied, respectively. We calculated 50% withdrawal thresholds, as described previously^80^.

### Sucrose preference test (SPT)

In the acclimation phase, animals drank two identical bottles (100 ml in each bottle) for 5 days: one contained 1% sucrose solution and the other contained water. For avoiding side preference, we alternated sides of the bottles every day. Before surgery, baseline sucrose preference was determined and only rats that displayed a sucrose preference ≥65% were included in this study^25^. During test phases 1 and 2, two bottles were provided for each animal for 24 h. At the end of each test, sucrose preference was calculated as volume of sucrose consumed divided by total liquid consumption for each rat.

### Forced swim test (FST)

The FST was applied as documented in the previous study^82^. The FST was conducted in a transparent plexiglas cylinder (65 cm height, 30 cm diameter) filled with water (23–24 °C) to a depth of 45 cm for 6 min. Water in the cylinder was changed for each rat. After test, the animal was taken out of the cylinder, dried and put back into its home cage. Immobility time during last 5 min of the 6-min test was calculated as floating in the water without struggling except making necessary movements to keep its head above water.

### Open field (OF)

On day 14 after surgery, rats were individually placed in the center of a gray polyvinyl chloride box (100 × 100 × 40 cm). Each rat was allowed to explore in the box for 5 min while the exploratory behavior and spontaneous motor activity were automatically recorded by a video tracking system.

### Western blot analysis

Rats were sacrificed after anesthetized with sodium pentobarbital (80 mg/kg) and the PFC tissues were collected on ice plate. Tissues were homogenated in RIPA buffer and protease inhibitors. Homogenate was centrifuged at 12000 rpm at 4 °C centrifuge for 15 min, and the supernatant was retained. The protein concentration was determined by Bradford method using bovine serum albumin as a standard. The normalized protein samples were separated on 12% SDS-PAGE and transferred to polyvinylidene fluoride membrane. Membranes were then blocked with 5% non-fat milk at room temperature for 1 h and incubated overnight at 4 °C with rabbit anti-BDNF (1:1000, Abcam, ab203573) and rabbit anti-GAPDH (1:5000, Millipore). Membranes were washed 3 × 10 min with PBST and incubated with secondary antibody (goat anti-mouse and goat anti-rabbit, Bioworld Technology) diluted in 1:5000 for 1 h at room temperature. After washing the membranes 3 × 10 min, bands were visualized using chemiluminescence quantitated with Image J software (Wayne Rasband, National Institute of Health, MA, USA).

### Enzyme-linked immunosorbent assay (ELISA)

Tissue levels of IL-1β, IL-6 and TNF-α in the PFC were quantified by the ELISA (n = 6 or 7 for each group, twice quantifications for each tissue) according to the protocol provided by the manufacturer (Jiancheng, China). Rats were killed by decapitation with the anesthesia of sodium pentobarbital (80 mg/kg), and the PFC tissues were collected. The tissue was homogenized with each 1 mg tissue added 9 uL saline. Homogenate was centrifuged for 10 min at 2500 rpm at 4 °C centrifuge, and the supernatant was obtained. Then each protein concentration was quantified by Bradford method. The standard curves for the cytokines were generated using Optical Density (OD) tested at 450 nm. The concentration of the cytokines was calculated by the standard curves.

### Statistical analyses

In the experiment 1, the data were standardized by z scores. Then, hierarchical cluster analysis was performed using Ward’s method and applying squared Euclidean distance as the distance measure, and rats were classified as “rats with depression-like phenotype” or “rats without depression-like phenotype” clusters.

In the experiment 2, a two-group discriminant function analysis was performed to predict the rats with or without depression-like phenotype. A discriminant function analysis compared the two groups defined by the previous hierarchical cluster analysis in the experiment 1. The canonical discriminant function coefficients can be seen as an indication of the contribution each variable makes to the equation. Based on the discriminant function scores, rats were classified as “ketamine responder group” or “ketamine non-responder group” clusters.

Data are presented as the mean ± standard error of the mean (S.E.M.). Repeated measured one-way ANOVA, followed by Tukey test was used to detect interactions between test time points and groups. Data of Western blot and ELISA were analyzed using one-way ANOVA, followed by the Tukey test. Pearson correlation analysis was performed to examine the correlation between the changes of sucrose preference after ketamine injection and serum levels of cytokines at baseline. Statistical analyses were performed using SPSS16.0, with a significance level of *P* < 0.05.

## Electronic supplementary material

Supplemental information
