# Supplementary material for: Alterations in the inflammatory cytokines and brain-derived neurotrophic factor contribute to depression-like phenotype after spared nerve injury: improvement by ketamine
Source: Sci Rep. 2017 Jun 9;7:3124. doi: 10.1038/s41598-017-03590-3 (PMC5466642; doi:10.1038/s41598-017-03590-3)

Supplemental Information

Alterations in the inflammatory cytokines and brain-derived neurotrophic factor contribute to depression-like phenotype after spared nerve injury: improvement by ketamine

Ze-Min Xie[1,2], Xing-Ming Wang[1], Ning Xu[2], Jing Wang[1,3], Wei Pan[3], Xiao-Hui Tang[1,3], Zhi-Qiang Zhou[1,3], Kenji Hashimoto[4]*, Jian-Jun Yang[1]*

[1]Department of Anesthesiology, Zhongda Hospital，Medical School, Southeast University, Nanjing, China

[2]Jiangsu Province Key Laboratory of Anesthesiology & Jiangsu Province Laboratory of Anesthetic and Analgesia Application Technology, Xuzhou Medicine University, Xuzhou, China

[3]Department of Anesthesiology, Jinling Hospital, School of Medicine, Nanjing University, Nanjing, China

[4]Division of Clinical Neuroscience, Chiba University Center for Forensic Mental Health, Chiba, Japan

*Correspondence and requests for materials should be addressed to Prof. Jian-Jun Yang (yjyangjj@126.com) or Prof. Kenji Hashimoto (hashimoto@faculty.chiba-u.jp)

Figure S1. SNI induced mechanical hyperalgesia and depression-like behaviors. **(a):** In the MWT, SNI surgery induced mechanical hyperalgesia compared with sham surgery. **(b):** SNI surgery induced less weight gain 14 days after surgery compared with sham surgery. **(c):** In the SPT, SNI surgery induced a reduction of sucrose preference compared with sham surgery. **(d):** In the SPT, SNI surgery did not alter total fluid consumption. **(e):** In the FST, SNI surgery induced increased immobility time 14 and 21 days after surgery compared with sham surgery. **(f):** In the OF, SNI surgery did not altered total distance. SNI group, n=32; Sham group, n=14. $^{\#}P < 0.05$, $^{\#\#}P < 0.01$ and $^{\#\#\#}P < 0.001$ vs sham group; $^{*}P < 0.05$ and $^{***}P < 0.05$ vs baseline.

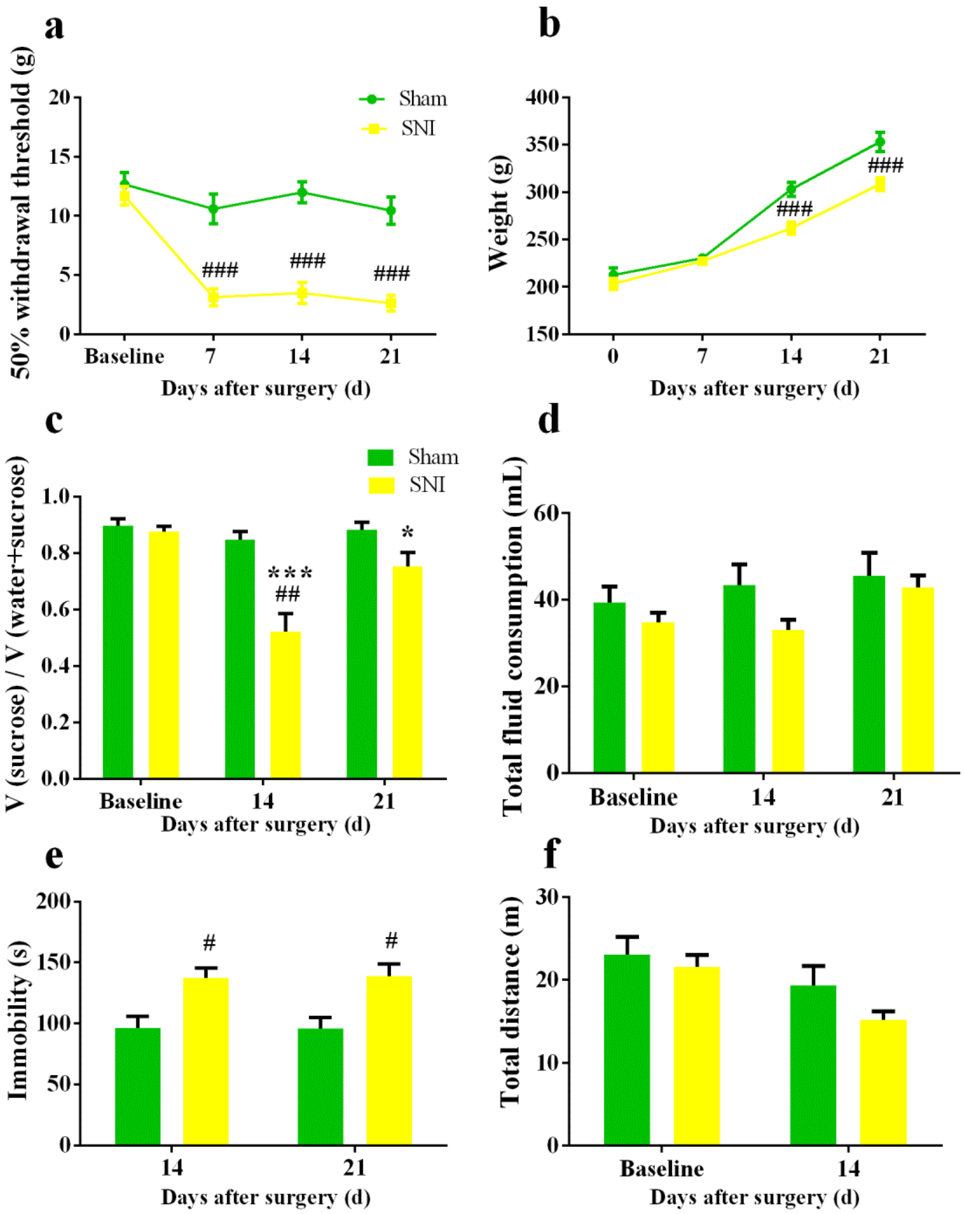

Figure S2. **Dendrogram depict the classification of SNI subjects.** As the dendrogram showed, SNI rats were statistically classified into two clusters by hierarchical cluster analysis. Cluster 1 (n=13) was regarded as “rats with depression-like phenotype” and cluster 2 (n=19) was “rats without depression-like phenotype”.

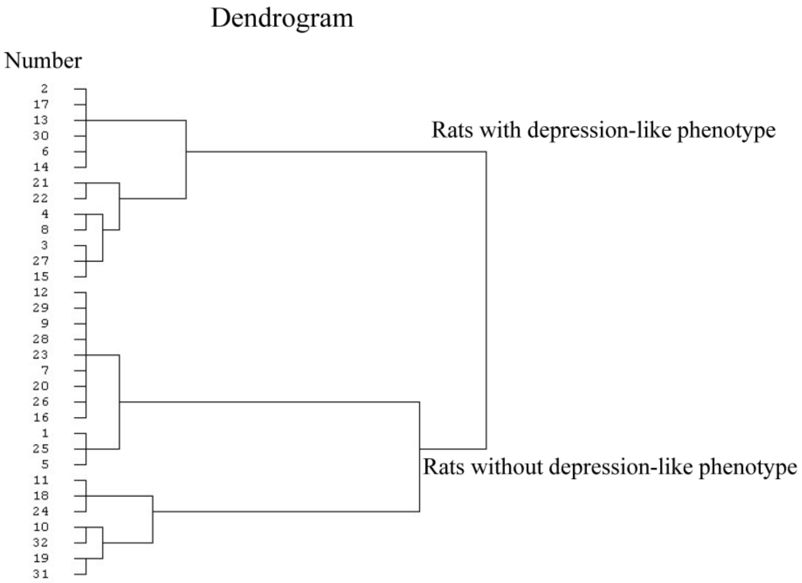

Fig. S3. The western blot of BDNF in the PFC after SNI surgery.

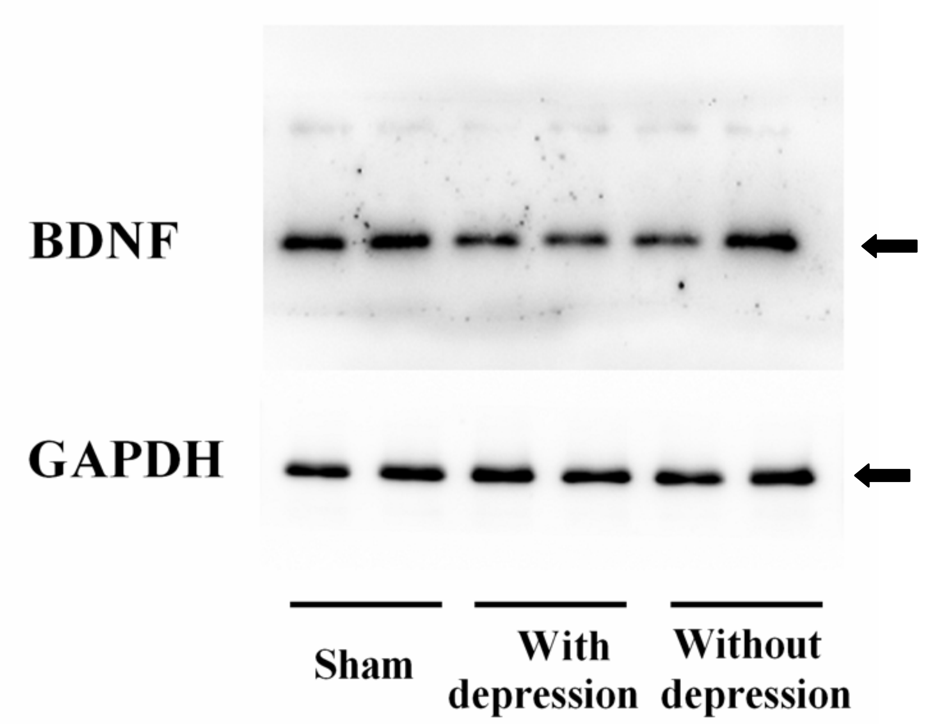

Supplement: Supplementary file 1 — Supplemental information [file 41598_2017_3590_MOESM1_ESM.pdf]
